# Supplementary material for: Uncovering transcriptional dark matter via gene annotation independent single-cell RNA sequencing analysis
Source: Nat Commun. 2021 Apr 12;12:2158. doi: 10.1038/s41467-021-22496-3 (PMC8042062; doi:10.1038/s41467-021-22496-3)
Supplement: Supplementary file 3 — Reporting Summary [file 41467_2021_22496_MOESM3_ESM.pdf]

nature research

Corresponding author(s): Iwijn De Vlaminck

Last updated by author(s): March 2, 2021

Reporting Summary

Nature Research wishes to improve the reproducibility of the work that we publish. This form provides structure for consistency and transparency in reporting. For further information on Nature Research policies, see our Editorial Policies and the Editorial Policy Checklist.

Statistics

For all statistical analyses, confirm that the following items are present in the figure legend, table legend, main text, or Methods section.

n/a	Confirmed	
☐	☒	The exact sample size (*n*) for each experimental group/condition, given as a discrete number and unit of measurement
☐	☒	A statement on whether measurements were taken from distinct samples or whether the same sample was measured repeatedly
☐	☒	The statistical test(s) used AND whether they are one- or two-sided<br>*Only common tests should be described solely by name; describe more complex techniques in the Methods section.*
☒	☐	A description of all covariates tested
☐	☒	A description of any assumptions or corrections, such as tests of normality and adjustment for multiple comparisons
☐	☒	A full description of the statistical parameters including central tendency (e.g. means) or other basic estimates (e.g. regression coefficient) AND variation (e.g. standard deviation) or associated estimates of uncertainty (e.g. confidence intervals)
☐	☒	For null hypothesis testing, the test statistic (e.g. *F*, *t*, *r*) with confidence intervals, effect sizes, degrees of freedom and *P* value noted<br>*Give P values as exact values whenever suitable.*
☒	☐	For Bayesian analysis, information on the choice of priors and Markov chain Monte Carlo settings
☒	☐	For hierarchical and complex designs, identification of the appropriate level for tests and full reporting of outcomes
☐	☒	Estimates of effect sizes (e.g. Cohen's *d*, Pearson's *r*), indicating how they were calculated

Our web collection on statistics for biologists contains articles on many of the points above.

Software and code

Policy information about availability of computer code

Data collection	Github repository: https://github.com/fw262/TAR-scRNA-seq<br><br>Publicly available datasets were downloaded directly from their respective repositories using fastq-dump v2.9.6 SRA tool.

Data analysis	The custom software leverages many publicly available tools listed below. Please refer to https://github.com/fw262/TAR-scRNA-seq for more information.<br>-Snakemake v5.20.0 (https://snakemake.readthedocs.io/en/stable/)<br>-Drop-seq Computational Tools v2.3.0 (https://github.com/broadinstitute/Drop-seq/releases)<br>-Picard Tools v2.18.29-0+ (https://broadinstitute.github.io/picard/)<br>-STAR Aligner v2.5+ (https://github.com/alexdobin/STAR/releases)<br>-R v3.5+ (https://www.r-project.org/)<br>-BiocManager v1.30.4 (https://cran.r-project.org/web/packages/BiocManager/vignettes/BiocManager.html)<br>-rtracklayer v1.42.2 (https://bioconductor.org/packages/release/bioc/html/rtracklayer.html)<br>-groHMM v1.16.0 (https://bioconductor.org/packages/release/bioc/html/groHMM.html)<br>-Seurat v3.2.1 (https://satijalab.org/seurat/articles/install.html)<br>-data.table v1.12.8 (https://github.com/Rdatatable/data.table)<br>-dplyr v1.0.2 (https://www.r-project.org/nosvn/pandoc/dplyr.html)<br>-stringr v1.4.0 (https://cran.r-project.org/web/packages/stringr/readme/README.html)<br>-Samtools v1.11+ (http://www.htslib.org/)<br>-gtfToGenePred v377-1+ (https://bioconda.github.io/recipes/ucsc-gtftogenepred/README.html)<br>-bedtools v2.29.2+ (https://bedtools.readthedocs.io/en/latest/)<br>-BLAST v2.9.0+ (https://blast.ncbi.nlm.nih.gov/Blast.cgi)

For manuscripts utilizing custom algorithms or software that are central to the research but not yet described in published literature, software must be made available to editors and reviewers. We strongly encourage code deposition in a community repository (e.g. GitHub). See the Nature Research guidelines for submitting code & software for further information.

Data

Policy information about availability of data

All manuscripts must include a data availability statement. This statement should provide the following information, where applicable:

- Accession codes, unique identifiers, or web links for publicly available datasets
- A list of figures that have associated raw data
- A description of any restrictions on data availability

Publicly available datasets: Fastq files for the human PBMC dataset were downloaded directly from the 10X Genomics library of single cell gene expression data. Tabula Muris alignment files (BAMs) for 10X Genomics droplet generated data were downloaded from the Gene Expression Omnibus (GSE109774) and fastq files were extracted using the 10X Genomics bamtofastq tool43. Mouse uTARs were generated based on combining alignment files across all droplet generated data and scRNA-seq analysis for kidney (10X_P4_5) and spleen (10X_P4_6) samples are shown. Fastq files for the naked mole rat and sea urchin datasets were downloaded from GEO listed in their respective publications (GSM3885302 and GSE134350 respectively). Naked mole rat uTARs were generated based on combining alignment files for the spleen samples (SRR9291380, SRR9291381, SRR9291382, SRR9291383, SRR9291384, SRR9291385, SRR9291386, SRR9291387) and scRNA-seq analysis for sample SRR9291380 (nmr_1.1) is shown. Sea urchin embryo uTARs were generate based on combining alignment files generated from SRR9693264, SRR9693265, and SRR9693266 and scRNA-seq analysis for sample SRR9693264 (D1) is shown. Gray mouse lemur lung tissue uTARs were generated from the Tabula Microcebus consortium by combining MLCA_ANTOINE_LUNG_EPCAM_POS_S12, MLCA_ANTOINE_LUNG_CD31_POS_S11, MLCA_ANTOINE_LUNG_P3_S7 datasets and scRNA-seq analysis for MLCA_ANTOINE_LUNG_EPCAM_POS_S12 is shown. Gray mouse lemur spleen tissue uTARs were generated from the Tabula Microcebus consortium from the MLCA_ANTOINE_SPLEEN dataset. Gray mouse lemur SS2 testes data was collected from the Tabula Microcebus consortium.

The chicken related sequencing data discussed in this publication have been deposited in NCBI's Gene Expression Omnibus and are accessible through GEO Series accession number GSE149457. H&E stained tissue images for spatial RNA-seq datasets have been made available through the Git repository. (https://github.com/fw262/TAR-scRNA-seq)

Field-specific reporting

Please select the one below that is the best fit for your research. If you are not sure, read the appropriate sections before making your selection.

☒ Life sciences ☐ Behavioural & social sciences ☐ Ecological, evolutionary & environmental sciences

For a reference copy of the document with all sections, see nature.com/documents/nr-reporting-summary-flat.pdf

Life sciences study design

All studies must disclose on these points even when the disclosure is negative.

Sample size	The number of cells used for single-cell analysis was determined based on thresholding for cells with a minimum number of genes and transcripts as to remove dropouts and doublet cells. We made sure to perform the same quality filtering for publicly available datasets as their original publication. They are described in the methods of the paper for each individual experiment.
Data exclusions	All data sets were included in the paper.
Replication	We replicated our analysis across several publicly available datasets that span multiple organisms, tissues, and sequencing platforms (short-read versus full-length sequencing).
Randomization	We perform comparative single cell RNA-sequencing analysis across multiple organisms and tissues. We did not perform randomization since direct comparison across the datasets was required.

Blinding	Blinding is not relevant to this study since we performed non-biased single-cell analysis using techniques without prior assumptions (e.g. PCA, UMAP). All data analyses were performed using automated software tools.

Reporting for specific materials, systems and methods

We require information from authors about some types of materials, experimental systems and methods used in many studies. Here, indicate whether each material, system or method listed is relevant to your study. If you are not sure if a list item applies to your research, read the appropriate section before selecting a response.

Materials & experimental systems

n/a	Involved in the study
☒	☐ Antibodies
☒	☐ Eukaryotic cell lines
☒	☐ Palaeontology and archaeology
☐	☒ Animals and other organisms
☒	☐ Human research participants
☒	☐ Clinical data
☒	☐ Dual use research of concern

Methods

n/a	Involved in the study
☒	☐ ChIP-seq
☒	☐ Flow cytometry
☒	☐ MRI-based neuroimaging

Animals and other organisms

Policy information about studies involving animals; ARRIVE guidelines recommended for reporting animal research

Laboratory animals	Bovans brown embryonic chickens (gallus gallus species) were incubated to the embryonic day of interest and hearts were isolated. Sex of the animal were not documented.
Wild animals	The study did not involve wild animals.
Field-collected samples	The study did not involve samples collected from the field.
Ethics oversight	Use of chick embryos at these developmental stages renders them outside of IACUC regulations. All embryos were quickly isolated and euthanized via decapitation.

Note that full information on the approval of the study protocol must also be provided in the manuscript.
